# A psychometric assessment of a network social capital scale among sexual minority men and gender minority individuals

**DOI:** 10.1186/s12889-021-11970-8

**Published:** 2021-10-22

**Authors:** Meagan Zarwell, Jennifer L. Walsh, Katherine G. Quinn, Andréa Kaniuka, Alexandra Patton, William T. Robinson, Robert J. Cramer

**Affiliations:** 1 Department of Public Health Sciences, University of North Carolina at Charlotte, Charlotte, North Carolina USA; 2 Center for AIDS Intervention Research, Medical College of Wisconsin, Milwaukee, WI USA; 3 School of Public Health, Louisiana State University Health Sciences Center, New Orleans, Louisiana USA

**Keywords:** Sexual minority men and gender minority individuals; social capital, Social networks, Social support

## Abstract

**Background:**

Social capital, the potential for individuals to access resources through group memberships, is linked to a constellation of health outcomes. We modified a previously evaluated Constructed Family Social Capital Scale for gay, bisexual and other men who have sex with men who belong to constructed families to create a new measure of social capital within sexual minority men and gender minority individuals’ social networks.

**Methods:**

Participants were recruited from a Pride festival in Milwaukee, Wisconsin in 2018 to complete a cross-sectional survey. This analysis is restricted to 383 participants who identified as sexual minority men or gender minority individuals and completed nine items measuring social capital within their social networks. We conducted exploratory and confirmatory factor analyses to assess factor structure. Internal consistency was measured using Cronbach’s *alpha*.

**Results:**

Reliability was high, indicating the scale’s utility to assess Network Social Capital among sexual minority men and gender minority individuals. A single-factor solution with high factor loadings was found for the nine-item scale.

**Conclusions:**

This study extended the psychometric properties of a preliminary social capital instrument modified from a prior study in a different population and context. The modified measure has implications for use among sexual minority men and gender minority individuals to measure social capital within social networks. Previous studies suggest that interventions to enhance social capital among sexual minority men and gender minority individuals may be beneficial for HIV prevention interventions. This tool may be relevant for the evaluation of social capital interventions within networks of sexual minority men and gender minority individuals.

## Introduction

The theory of social capital argues that social connections and networks are valuable to members of groups who may derive resources from within their networks [1–4]. The two dominant perspectives of social capital, the network approach and social cohesion approach [3], are distinct in several ways. Network approaches employ social network analyses and typically measure resources proffered from network membership [5–8], whereas a social cohesion approach measures social capital as trust, reciprocity, civic engagement, and social participation [9–12]. Similarities in the two dominant perspectives include their embeddedness within an ecological framework, whereby an individual’s health and behavior are influenced from factors at multiple levels. Despite differences in measurement, both social capital perspectives define social capital as the resources afforded by social connections and the potential for an individual to gain access to those resources. Few studies have integrated network measures with social cohesion to measure social capital [1, 12].

Higher social capital may be protective for multiple health outcomes, including HIV infection [13, 14]. Sexual minority men and gender minority individuals, including gay, bisexual, and other men who have sex with men (GBMSM) and transgender people of color, experience the highest rates of new HIV diagnoses among all groups. An estimated one in two Black GBMSM and one in four Latino GBMSM will acquire HIV in their lifetime [15] and one in six GBMSM living with HIV is unaware of their status [16]. The HIV burden among trans women is startling: the estimated prevalence of HIV is 34 times that of cisgender adults [17, 18], and incident infections among trans women remain high [19]. Among US adults, less than 0.5% are living with HIV infection. However, a recent metanalysis reported that overall HIV prevalence among transgender individuals is 9.2%, and the prevalence among trans women is even higher (14.1%). HIV prevalence among trans men is approximately 3.2% [20]. Trans people of color are disproportionately affected by HIV: 44% of trans individuals diagnosed are Black and 26% are Hispanic/Latinx [18].

Recent US studies have explored social capital and HIV outcomes. In Los Angeles, a study found that HIV transmission risk behaviors and HIV testing were associated with higher social capital resources [21]. Ransome et al. [22] found that higher social capital was associated with lower odds of concurrent sexual partnerships among African American women compared to men. Men with higher social capital were more likely to engage in concurrent sexual relationship. Another study found that social capital moderated the relationship between sex-work-related stigma and condomless sex acts with non-paying partners. The association was significant among male sex workers with lower social capital, but not among men with higher social capital [23]. Another study found that loss of social capital within family and social relationships motivated GBMSM to not disclose their sexual orientations or identities [24]. Measures of social capital have also been associated with HIV medication adherence among people living with HIV [25]; late HIV diagnoses [26, 27], and STI diagnoses [28]. International studies have employed several different measures of social capital and found associations with higher rates of HIV testing among GBMSM [29], reduced risk behaviors and participation in HIV-related meetings among female sex workers [30], and reduced HIV risk behaviors and a decline in HIV incidence [31, 32].

Zarwell and Robinson established a preliminary instrument, the *Constructed Family Social Capital Scale*, that blends social cohesion and network indicators to measure social capital among GBMSM who belonged to constructed families [33]. They argued that social capital is a collective construct created through participation in social organizations, such as constructed families, which may be characterized by network indicators**.** Constructed families are important social networks within the lesbian, gay, bisexual, transgender, queer, and questioning (LGBTQ) community and include gay families, pageant families, and the house ball community [34–38]. Constructed families are an important source of social support within the LGBTQ community and membership within constructed families has been associated with lower risk behaviors among GBMSM of color. The social embeddedness of constructed family members supports both of the dominant perspectives of social capital. Constructed families offer peer support to sexual minority men and gender minority individuals who may experience intersectional stigmas related to their racial and gender identities or sexual orientation, which may promote health behaviors including HIV testing, medication to prevent HIV infection, and treatment for people living with HIV [38–41].

In this exploratory study, we assess the previously evaluated *Constructed Family Social Capital Scale* to create a new tool to measure social capital within social networks of sexual minority men and gender minority individuals.

## Methods

### Participants and setting

In June 2018, we recruited sexual minority men and gender minority individuals attending a Pride Festival in Milwaukee, a mid-sized city the Midwestern U.S., to complete an anonymous paper survey. Study staff and signage recruited potential participants walking past a booth in the Health and Wellness area at the event. Eligibility included individuals aged 18 or older, able to complete the survey in English, who identified as cisgender sexual minority men or a reported a gender identity that did not align with their sex assigned at birth (i.e., transgender, non-binary, or another identity). All methods were carried out in accordance with relevant guidelines and regulations. The Institutional Review Board at the Medical College of Wisconsin approved the study protocols. Informed consent was obtained from all participants. Potential participants were given an informational letter explaining the study purpose and risks and benefits to participation prior to completing the survey, which indicated consent to participate. Participant incentives included a choice between five dollars or a small item of equal value. Participants were also offered flyers with information about local resources (e.g. free HIV testing, and prevention services, etc.). The social capital items were completed by 386 out of the total 415 survey participants. Of these, three cisgender men were excluded from these analyses because they identified as “straight”. Thus, the final analytic sample size was 383 (see Table 1 in the results for demographic information).

### Measures

#### Demographics

*Race* was categorized as Black, White, or another race. *Ethnicity* measured whether participants identified as Latinx/Hispanic. *Gender* was measured using a two-step process of assessing sex assigned at birth and current gender identity [42]. Participants were first asked to report their sex assigned at birth and the response options included male, female, and intersex. Gender was assessed by asking “What is your current gender identity”. Response options included: Male, female, trans man, trans woman, non-binary, and another identity. Respondents who selected “another identity” and who indicated a non-binary or gender fluid identity were recoded as “non-binary”. Participants assigned male sex at birth who identified as female were categorized as “trans women” and participants assigned female sex at birth who selected male were categorized as “trans men.” No participants selected “Intersex” for sex assigned at birth. Participants whose sex assigned at birth aligned with their current gender identity were categorized as cisgender men. *Work status* was measured using three categories: full time, part time, or unemployed. *Sexual orientation* categories included gay, bisexual, straight, or another identity. *Material hardship* was measured by summing and averaging five items that assessed how frequently participants were unable to afford to pay for their basic needs (bills, food, leisure, clothing, and medical care) [43]. On a scale of 1 to 5, a higher score indicates greater material hardship.

#### Social Capital

We modified a previously published preliminary instrument that measured social capital within constructed families of GBMSM (i.e., men who belonged to a gay family, pageant family, or the house ball community). The original *Constructed Family Social Capital Scale* asked men about members of their constructed families. The original scale included the following domains of social capital among GBMSM in constructed families: social influence, multiplex ties, heterogeneity, social cohesion, trust, quality of support, and compositional quality [33]. Several of these measures reflect tie strength, which may influence social regulation in a network (strong ties) or link people across multiple social spheres (weak ties). Tie strength may influence the level of relational support of an individual within a network, including the ease of obtaining external information or resources [1]. *Social influence* refers to the number of network members who influence peer norms and decision making which may regulate group members behaviors. *Multiplexity*, a measure of tie strength, refers to multi-purpose ties such as the number of network members who are simultaneously a friend and a colleague, which may result in stronger ties and greater social support [44, 45]. *Heterogeneity*, a measure of the diversity of attributes of group members, refers to variations in socio-demographic indicators within the network members such as differences by race, gender, sexual orientation, and age [1]. *Trust* is an aggregate measure of the trustworthiness among connections within a network [4]. *Quality of support*, another measure of tie strength, measures the number of group members who provide different forms of social support (e.g. emotional, instrumental, or informational support) [46]. *Social cohesion* assesses the context of social bonds within the group, and refers to feelings of belonging or inclusion among members [3, 45]. *Compositional quality* is indicated by the number of members an individual may be able to ask for specific advice or assistance with issues that may be important to members (in this case, health-related) [1, 47].

The original *Constructed Family Social Capital Scale* included eight items that measured social influence, tie strength (multiplex ties and quality of support), heterogeneity, trust, social cohesion, and compositional quality (advice about general and sexual health). In the original study, two items measuring heterogeneity and social cohesion were removed. The exploratory factor analysis resulted in a final *Constructed Family Social Capital Scale* with six highly correlated items exhibiting high factor loadings (α = 0.84).

Our modified version, the *Network Social Capital Scale,* asks the same questions in reference to members of social support networks among gender and sexual minority individuals. That is, the modified questions refer to members of one’s social network, instead of asking specifically about constructed families. We included all eight of the original items and added an additional item to assess the number of people within one’s social network that a participant could go to for advice or information about LGBTQ-related health care. This additional measure of compositional quality was added because sexual minority men and gender minority individuals face unique barriers to health care and discrimination in health care settings due to their marginalized identities [48–50] and therefore may benefit from social network members who they can talk to about LGBTQ-related healthcare. In addition, we modified phrasing of the social cohesion item such that the response options did not need to be reversed scored. Thus, all nine measures’ response options were aligned in the same direction and elicited information about participants’ social networks. The full list of questions in our modified scale is displayed in the results in Table 2.

#### Social network size

 One question assessed a baseline count of each participant’s social network size, “*About how many people are in your social network (i.e., people you hang out with who provide you with resources or social support)?*” The series of nine social capital items were asked in reference to the number of people within each individual’s social network.

### Data analysis

Three individuals were dropped from analyses because they identified as heterosexual cisgender men, thus violating criteria to complete social capital items. This sample adjustment yielded a final analyzable sample size of 383. SPSS v. 26 was used for handling missing data, derivation of subsamples, internal consistency, exploratory factor analyses (EFA), and independent sample t-tests. AMOS v. 26 was used for confirmatory factor analyses (CFA). Survey response data were used for those with responses to any valid responses to the social network and social capital items (*N* = 383). Among this sample, data missingness ranged from 7.5 to 16.1%. Missing social capital items were supplanted via multiple imputation [51].

For exploration of our *Network Social Capital Scale* factor structure, the full sample was split into two random subsamples, one for EFA (*n* = 185) and one for CFA (*n* = 198). Sample sizes satisfy requirements for EFA [52] and CFA [53], respectively. Based on statistical literature guidelines [52], EFA specification featured maximum likelihood estimation and promax rotation (as any subscales were expected to correlate). A combination of eigenvalues, scree plot, and factor loadings were inspected to determine the ideal number of factors. CFA specification featured maximum likelihood estimation with inspection of modification indices with an index over 10. Model fit interpretation was guided by inspection of the following fit indices: comparative fit index (CFI), Tucker-Lewis Index (TLI), Root Mean Square Error of Approximation (RMSEA), Standardized Root Mean Square Residual (SRMR), and *χ*^*2*^. Fit determination was assessed using established cut-scores in the literature [54, 55]. Internal consistency values are reported as Cronbach’s *alpha*. One EFA model was run using all nine *Network Social Capital Scale* items. Two CFA models were conducted: one set using the modified six items supported from the *Constructed Family Social Capital Scale* [33], and one set using all nine items supported by our EFA results.

Following the previously evaluated *Constructed Family Social Capital Scale* [33], the *Network Social Capital* items were tabulated as a proportion of number of persons listed for that item divided by one’s total social network size. Participants provided a count response to each of the nine social capital items for the number of people within their social network. Using the social network size as the denominator, we created proportions for each item. The proportions of all of the social capital measures were then averaged for a final social capital score. Bivariate analyses compared the average social capital scores by demographic characteristics.

## Results

In total, 298 participants were cisgender men (77.8%), 35 were trans men (9.1%), 27 individuals identified as non-binary or another identity (7.0%), and 23 participants were trans women (6.0%). The majority of the sample were White (*n* = 235; 61.5%), followed by Black (*n* = 97; 25.1%), or more than one or another race (*n* = 51; 13.1%). Only 42 participants (11.3%) were Hispanic/Latinx. Sixty-nine (17.9%) participants identified as bisexual, 266 (68.9%) identified as gay, 19 (4.9%) identified as pansexual, 15 (3.9%) identified as straight, and 17 (4.4%) reported another sexual identity. None of the cisgender men identified as straight. In total, 228 (59.5%) participants worked full-time, whereas 91 (23.6%) reported working part-time and 64 (16.7%) were unemployed. Participant ages ranged from 18 to 97, with a mean age of 31.75 years (*SD* = 13.38). Participants reported moderate material hardship on average, with a mean score of 2.52 (*SD* = 1.36). On average, participants reported a social network size of 17.13 people (*SD* = 40.91, Range: 0–500).

**Table 1:** Demographic Characteristics (*N* = 383)

	Total	
	N	%
**Gender Identity**		
Man	298	77.8
Trans man	35	9.1
Trans woman	23	6.0
Non-binary or another identity	27	7.0
**Race**		
Black	97	25.1
White	235	61.5
More than one race or Another race	51	13.1
**Ethnicity**		
Hispanic / Latinx	42	11.3
**Sexual Orientation**		
Bisexual	69	18.0
Gay	266	69.2
Pansexual	19	5.0
Straight	12	3.13
Other	17	4.4
**Work Status**		
Full-time	228	59.5
Part-time	91	23.8
Unemployed	64	16.7

### Factor analyses

EFA results suggested meaningful factor structure among *Network Social Capital Scale* items (KMO = .85; Bartlett’s Test *χ*^*2*^ [35] = 839.62, *p* < .001). The scree plot suggests a maximum of two factors, and eigenvalue cut-offs for factor 1 (eigenvalue = 4.34, 48.24% variance explained) and factor 2 (eigenvalue = 1.20, 13.33% variance explained) supports this maximum of two factors. Using a factor loading cut-off of .30, all items cross-load on both factors. Such high degrees of cross-loading support the retention of only one factor [52], in this instance comprising a total social capital score. Internal consistency for this total score was acceptable (α = .86). Factor loadings are listed in Table 2.

The *Network Social Capital Scale* demonstrated acceptable fit to the data, χ^*2*^ (21) = 44.93, *p* = .006, CFI = .97, SRMR = .04, TLI = .96, RMSEA = .07 (90% CI = .04, .10). This included three pairs of theoretically-supported correlated error terms (1 and 2; 4 and 6; 8 and 9). All items demonstrated expected significant positive loadings on the social capital latent variable (see Table 2, all *p*s < .001). The original six items in the *Constructed Family Social Capital Scale* demonstrated acceptable fit to the data, χ^*2*^ (13) = 22.24, *p* = .004, CFI = .97, SRMR = .04, TLI = .94, RMSEA = .10 (90% CI = .05, .15). This included one pair of correlated theoretically-supported error terms (1 and 2), as suggested by modification indices. All items demonstrated expected significant positive loadings on the social capital latent variable (see Table 2, all *p*s < .001). Internal consistency was acceptable both for the total score using all nine items (α = .88) and the shortened set of six items modified from the *Constructed Family Social Capital Scale* (α = .83). We found no statistically significant differences in social capital scores based on demographic characteristics.

**Table 2:** Factor Analysis Item Loadings for Network Social Capital Scale

Out of all the people in your social network, about how many …	Average of Proportion	EFA F1	EFA F2	CFA: EFA Model	CFA: CFSC
1. (Social Influence) Have influenced important decisions in the past 3 months?	0.38	.32	.52	.46	.45
2. (Multiplexity) Fulfill multiple roles in your life (i.e. a friend but also a classmate, co-worker, etc.)?	0.41	.32	.55	.40	.42
3. (Heterogeneity) Are similar to you (in terms of gender, race, sexuality, etc.)?	0.53	.39	.36	.59	–
4. (Trust) Do you trust in general?	0.63	.52	.77	.67	.68
5. (Quality of Support) Can you go to for advice or borrow money or something valuable if you need it?	0.48	.58	.76	.72	.72
6. (Social Cohesion) Would not take advantage of you if they got the chance?	0.60	.42	.66	.57	–
7. (Compositional Quality) Could you ask for advice or help about your health?	0.59	.83	.66	.89	.89
8. (Compositional Quality) Could you ask for advice or help about HIV or other STDs?	0.51	.95	.58	.82	.82
9. (Compositional Quality) Could you ask for advice or help about LGBTQ-related healthcare?	0.49	.86	.50	.78	–

*EFA* Exploratory Factor Analysis, *CFA* Confirmatory Factor Analysis, *F1* Factor 1, *F2* Factor 2, *EFA Model* Network Social Capital Scale (nine items), *CFSC* Constructed Family Social Capital Scale (original six items)

## Discussion

We tested a modified social capital scale originally developed for use within constructed families of GBMSM within a wider population of sexual minority men and gender minority individuals. The original six items from the *Constructed Family Social Capital Scale* performed well within our population of sexual minority men and gender minority individuals when asked in reference to social network members (α = .84). Moreover, the reliability for all nine of the modified items in our *Network Social Capital Scale* had high internal consistency (α = .87). Our exploratory and confirmatory factor analyses support the use of our *Network Social Capital Scale* items to measure social capital within social networks of sexual minority men and gender minority individuals. Whereas the original scale study participants were recruited from venues in New Orleans and items specifically asked questions about members of constructed families, we modified the questionnaire to ask about members of participants’ social support networks more broadly. Thus, our findings indicate that the original items asked in the *Constructed Family Social Capital Scale* function well in different settings and with different populations.

Researchers have long argued that harnessing networks may improve effective health promotion programs, particularly to reduce the spread of sexually transmitted infections such as HIV [56]. Recent approaches to address HIV disparities include increasing the uptake of pre-exposure prophylaxis (PrEP), a daily pill that effectively prevents sexual transmission of HIV, among sexual and gender minority individuals at elevated risk for HIV infection [57]. Studies have also found associations with social capital and awareness and willingness to take PrEP. For example, social capital measured as community group participation has been associated with awareness of and willingness to take PrEP among GBMSM [58, 59]. One study exploring resilience, resources, and networks concluded that family-based social capital or social support interventions may improve PrEP uptake among young Black GBMSM and trans women [60]. Together, these findings indicate the need for interventions to increase social capital among underserved populations at elevated risk for HIV infection, which may impact the HIV-prevention continuum, particularly the uptake of PrEP to prevent HIV infection [61, 62].

Our findings further our ability to measure social capital within social networks of sexual minority men and gender minority individuals to improve health promotion programming. The resources afforded to sexual minority men and gender minority individuals within their social networks may influence HIV [14] or other health outcomes, and this scale may be useful for future studies to explore the influence of social capital on HIV prevention and treatment interventions that leverage online networks such as Empowering with PrEP (E-PrEP) [63]. Given that social networks often share similar risk behaviors [64] and may vary by race or gender [65, 66], social network tools may be critical to rapidly identify HIV cases as they continue to disproportionately affect GBMSM and transgender women. Previous studies indicate that disparities in HIV within these disproportionately affected groups may be attributed to higher underlying prevalence of HIV within their networks [67], rather than greater risk behavior [41, 68–72], indicative of the need for social network interventions that harness networks for HIV prevention efforts [73]. A recent publication titled “A new era of HIV risk: It’s not what you know, it’s who you know (and how infectious)” suggests that future approaches to end HIV must take into account social, sexual, and drug use network connections [74]. Because network characteristics, including the degree of similarity (i.e. *homophily*), norms, and beliefs are important facilitators to HIV acquisition, future studies may adapt or use this scale to measure social capital within social, sexual, or drug using networks. In addition, researchers may utilize or modify this tool to measure and evaluate the efficacy of interventions designed to enhance social capital among sexual minority men and gender minority individuals.

### Limitations

Our cross-sectional study provides a snapshot of social capital measured among a population of patrons at a Pride festival event in Milwaukee in 2018, and therefore is not representative of all sexual minority men and gender minority individuals. It is possible that individuals with higher social capital and more community connectedness may attend Pride events. We assessed a previously developed preliminary instrument, which we modified to be more inclusive by adding an additional item about the number of social network members participants could talk to about LGBTQ health-related information. These limitations notwithstanding, our results provide a brief and reliable measure to assess social capital among sexual minority men and gender minority individuals. Our findings support wider use of this scale in larger samples using different recruitment methods, as venue or event-based recruitment may bias the applicability of this scale to individuals who are not reached by these methods.

## Conclusions

We investigated a psychometric assessment of social capital within social networks of sexual minority men and gender minority individuals. Our blended social capital scale incorporates measures of social cohesion and network indicators that may be useful to improve our understanding of social connections that impact health outcomes within social networks of sexual minority men and gender minority individuals. Future studies may use this scale within network studies and to evaluate social capital interventions.

## Data Availability

The datasets used and/or analysed during the current study are available from the corresponding author on reasonable request with a data use agreement.

## Abbreviations

GBMSM: Gay, Bisexual, and Other Men Who Have Sex with Men
PrEP: Pre-Exposure Prophylaxis
EFA: Exploratory Factor Analysis
CFA: Confirmatory Factor Analysis
CFI: Comparative Fit Index
TLI: Tucker-Lewis Index
RMSEA: Root Mean Square Error of Approximation
SD: Standard Deviation
